# Interstitial Lung Disease and Cardiotoxicity Associated with Trastuzumab Deruxtecan, Sacituzumab Govitecan, and Trastuzumab Emtansine: A Narrative Review

**DOI:** 10.3390/medicina62091664

**Published:** 2026-08-30

**Authors:** Raul Tirinescu, Ana-Maria Pah, Adina Tirinescu, Diana-Maria Mateescu, Camelia-Oana Muresan

**Affiliations:** 1 Department of Medical Oncology, M Hospital, 10–12 Witting Street, 010903 Bucharest, Romania; tirinescuraul@gmail.com; 2 Department of Cardiology, “Victor Babeș” University of Medicine and Pharmacy of Timișoara, Eftimie Murgu Square 2, 300041 Timișoara, Romania; 3 Department of Infectious Diseases, Clinical Hospital CF Timișoara, 1 Gării Street, 300166 Timișoara, Romania; diana.mateescu@umft.ro; 4 Institute of Legal Medicine Timișoara, 1A Cireșului Street, 300610 Timișoara, Romania; muresan.camelia@umft.ro; 5 Ethics and Human Identification Research Center, “Victor Babeș” University of Medicine and Pharmacy of Timișoara, Eftimie Murgu Square 2, 300041 Timișoara, Romania; 6 Discipline of Forensic Medicine, Bioethics, Deontology, and Medical Law, Department of Neuroscience, “Victor Babeș” University of Medicine and Pharmacy of Timișoara, Eftimie Murgu Square 2, 300041 Timișoara, Romania

**Keywords:** antibody–drug conjugates, trastuzumab deruxtecan, trastuzumab emtansine, sacituzumab govitecan, interstitial lung disease, pneumonitis, cardiotoxicity, left ventricular dysfunction, cardio-oncology

## Abstract

*Background and Objectives*: Antibody–drug conjugates (ADCs) have become a major therapeutic platform in breast cancer and other solid tumors. Trastuzumab deruxtecan (T-DXd), trastuzumab emtansine (T-DM1), and sacituzumab govitecan (SG) differ substantially in antibody target, linker, payload, drug-to-antibody ratio, and bystander effect, resulting in heterogeneous pulmonary and cardiac toxicity profiles. This narrative review critically compares interstitial lung disease (ILD)/pneumonitis and cardiotoxicity associated with these three agents, aiming to prevent inappropriate extrapolation of toxicity algorithms and to provide a practical, agent-specific framework for multidisciplinary care. *Materials and Methods*: A targeted narrative search of PubMed/MEDLINE, Google Scholar, ClinicalTrials.gov, regulatory product information, and oncology/cardio-oncology guidance was performed and updated on 24 August 2026. Priority was given to regulatory documents, pivotal trials, pooled safety analyses, real-world cohorts, systematic reviews, and multidisciplinary recommendations. Pharmacovigilance data and case reports were included only to characterize rare events. *Results:* T-DXd is associated with a clinically important ILD/pneumonitis risk (approximately 12–15% in pooled analyses), predominantly grade 1–2 but occasionally fatal, requiring proactive surveillance, immediate interruption for suspected disease, and grade-directed corticosteroid therapy. T-DM1 shows a low but established pneumonitis incidence of approximately 1%, with permanent discontinuation recommended upon diagnosis. SG-related pneumonitis is rare and incompletely defined, without a T-DXd-like surveillance mandate. Both T-DM1 and T-DXd retain trastuzumab-derived cardiac monitoring requirements; symptomatic heart failure remains uncommon, although protocol-defined LVEF declines appear more frequent with T-DXd. SG lacks an established cardiomyopathy signal. *Conclusions*: Cardiopulmonary toxicity of ADCs is agent-specific rather than a class effect. Monitoring intensity, diagnostic thresholds, and management pathways must be tailored to the individual drug, regimen, indication, dose, patient comorbidity, and prior therapy. Close collaboration among oncology, radiology, pulmonology, and cardio-oncology is essential to preserve both treatment efficacy and patient safety.

## 1. Introduction

Antibody–drug conjugates (ADCs) have become a major therapeutic platform in breast cancer and an expanding range of solid tumors. By linking a tumor-directed monoclonal antibody to a cytotoxic payload, ADCs aim to increase intratumoral drug delivery while reducing nonspecific systemic exposure. This concept is clinically successful but biologically incomplete: the antibody, target antigen, linker, payload, drug-to-antibody ratio (DAR), internalization kinetics, extracellular payload release, membrane permeability, and patient-specific factors jointly determine both efficacy and toxicity [1,2,3]. Consequently, adverse effects cannot be assumed to represent a uniform “ADC class effect”.

Trastuzumab emtansine (T-DM1), trastuzumab deruxtecan (T-DXd), and sacituzumab govitecan (SG) illustrate this heterogeneity. T-DM1 combines trastuzumab with the microtubule inhibitor DM1 through a stable, non-cleavable linker and has a mean DAR of approximately 3.5. T-DXd uses a trastuzumab-based antibody, a cleavable tetrapeptide linker, and a membrane-permeable topoisomerase I inhibitor payload, with a substantially higher DAR of approximately 8 and a clinically relevant bystander effect. SG targets trophoblast cell-surface antigen 2 (TROP-2) and delivers SN-38 through a hydrolysable linker, also with a high DAR [2,3,4,5,6]. These structural differences help explain why pulmonary and cardiac risks differ substantially among the three agents.

Interstitial lung disease (ILD)/pneumonitis is the defining serious toxicity of T-DXd. Although most events are grade 1 or 2, severe and fatal cases occur, and delayed recognition can permit rapid deterioration. The current United States prescribing information retains a boxed warning and requires permanent discontinuation for grade 2 or higher ILD/pneumonitis [7]. T-DM1 has a much weaker but recognized pulmonary signal; current regulatory information reports pneumonitis in approximately 1% of treated patients in major metastatic and adjuvant datasets and recommends permanent discontinuation when ILD/pneumonitis is diagnosed [8]. By contrast, ILD is not an established dominant toxicity of SG. Pneumonitis has been reported rarely in trials, pharmacovigilance analyses, and case reports, but the signal is substantially smaller and more uncertain than with T-DXd [9,10,11,12,13].

Cardiac toxicity also requires an agent-specific interpretation. T-DM1 and T-DXd retain a trastuzumab-derived antibody backbone and are used in populations frequently exposed to anthracyclines, trastuzumab, pertuzumab, radiotherapy, and other cardiovascular risk modifiers. Both agents therefore carry label-directed left ventricular ejection fraction (LVEF) assessment and treatment-modification recommendations [7,8]. Available pooled evidence suggests a low incidence of clinically important cardiac dysfunction with T-DM1 and a somewhat higher incidence of protocol-defined LVEF decline with T-DXd, while symptomatic heart failure remains uncommon [14,15,16,17]. SG is not HER2-directed and does not have an established cardiomyopathy signal; cardiovascular events observed during SG treatment must be interpreted in the context of advanced cancer, prior cardiotoxic therapy, anemia, infection, dehydration, electrolyte imbalance, infusion reactions, and concomitant drugs [9,11,12].

These three ADCs were selected because each has established clinical use in breast cancer and together they permit a clinically informative, mechanism-based comparison. T-DM1 and T-DXd share a trastuzumab-derived HER2-directed antibody but differ substantially in linker stability, payload, DAR, membrane permeability, and bystander effect, whereas SG provides a non-HER2-directed, TROP-2-targeted comparator with a topoisomerase I payload and a different safety profile. The selection therefore contrasts shared antibody biology, distinct ADC engineering, and different levels of maturity of pulmonary and cardiac safety evidence; it is not intended as an exhaustive review of every approved or investigational ADC.

This narrative review critically compares ILD/pneumonitis and cardiac toxicity associated with T-DXd, T-DM1, and SG. It integrates pivotal trials, pooled analyses, real-world cohorts, pharmacovigilance studies, case reports, regulatory information, and multidisciplinary guidance. The aims are to describe biological plausibility, incidence, severity, temporal patterns, risk modifiers, diagnosis, monitoring, management, treatment interruption, discontinuation, and rechallenge; to prevent inappropriate extrapolation of a toxicity algorithm from one ADC to another; and to provide a practical framework for multidisciplinary care.

## 2. Literature Identification and Scope

A targeted narrative literature search was conducted in PubMed/MEDLINE and supplemented by searches of Google Scholar, reference lists of key reviews and consensus papers, ClinicalTrials.gov records, regulatory product information, and official oncology and cardio-oncology guidance. The primary search window was 1 January 2010 to 24 August 2026; earlier seminal sources could be retained when required for mechanistic or historical context. The search was last updated on 24 August 2026. Search concepts combined the generic and development names of trastuzumab deruxtecan, trastuzumab emtansine, and sacituzumab govitecan with “antibody-drug conjugate”, “interstitial lung disease”, “ILD”, “pneumonitis”, “pulmonary toxicity”, “cardiotoxicity”, “left ventricular ejection fraction”, “heart failure”, “cardiomyopathy”, “arrhythmia”, “QT”, “real-world”, “pharmacovigilance”, “monitoring”, “management”, “combination”, and “rechallenge”.

A representative PubMed strategy was: ((“trastuzumab deruxtecan” [Title/Abstract] OR “DS-8201” [Title/Abstract] OR “trastuzumab emtansine” [Title/Abstract] OR “T-DM1” [Title/Abstract] OR “sacituzumab govitecan” [Title/Abstract]) AND (“interstitial lung disease” [Title/Abstract] OR pneumonitis [Title/Abstract] OR cardiotoxicity [Title/Abstract] OR “left ventricular ejection fraction” [Title/Abstract] OR “heart failure” [Title/Abstract] OR arrhythmia* [Title/Abstract] OR “QT prolongation” [Title/Abstract])). The strategy was adapted for each supplementary source.

Eligible evidence included current regulatory documents; randomized, pivotal, and other prospective clinical trials; prespecified or pooled safety analyses; observational cohorts reporting agent-specific pulmonary or cardiac outcomes; systematic reviews and meta-analyses; and multidisciplinary clinical guidance. Nonclinical studies were used only for mechanistic context. Pharmacovigilance studies and case reports were included to characterize rare or emerging presentations but were not used to calculate incidence because spontaneous reporting lacks a valid denominator and is vulnerable to stimulated reporting, duplication, missing data, and confounding by indication. Duplicate reports, non-substantive commentaries, and sources without agent-specific cardiopulmonary information were excluded. Conference abstracts were considered only when clinically relevant information was not yet available in a full peer-reviewed report and were identified as preliminary. No language filter was applied at the database-query stage; synthesis was limited to reports for which sufficient English-language information was available for reliable extraction and interpretation. Evidence from breast cancer was emphasized because it provides the largest clinical experience with all three ADCs; relevant gastric, lung, urothelial, and tumor-agnostic data were incorporated when they informed mechanisms or safety.

Records were managed at the level of the study family rather than counted as independent evidence whenever multiple publications reported overlapping populations from the same clinical trial. Duplicate records were removed, and the most mature or methodologically complete report was prioritized for incidence and safety estimates; companion publications were retained only when they contributed distinct follow-up, adjudication, subgroup, imaging, or management information. Because this was a narrative review, study selection was not conducted through a formal duplicate-screening workflow or a prespecified adjudication algorithm. Candidate sources and potentially discordant interpretations were discussed within the author group, and final inclusion and interpretation were agreed by consensus.

At the final update, 58 sources were cited. Of these, approximately 22 provided T-DXd-specific information, 13 provided T-DM1-specific information, and 14 provided SG-specific information. These agent-level categories overlap because regulatory documents, comparative trials, reviews, and guidance papers may address more than one ADC. The remaining sources informed ADC pharmacology, general drug-induced ILD, CTCAE grading, or cardio-oncology principles.

This article is a narrative review rather than a systematic review or meta-analysis. No protocol registration, PRISMA flow diagram, formal risk-of-bias instrument, or quantitative pooling was undertaken. Nevertheless, the search concepts, evidence hierarchy, and principal evidence sources are reported to improve transparency and reproducibility. Incidence estimates are presented with their source population, dose, indication, exposure, and method of event ascertainment whenever possible, because cross-trial comparisons without these qualifiers can be misleading.

For critical interpretation, signals were described narratively as well established, probable, or hypothesis-generating. A signal was considered well established when supported by regulatory recognition together with consistent prospective, pooled, or adjudicated evidence; probable when supported by repeated clinical observations and biological plausibility but limited by inconsistent definitions, small samples, or incomplete adjudication; and hypothesis-generating when derived mainly from spontaneous reports, disproportionality analyses, isolated cases, or noncomparative observations. These categories are interpretive descriptors rather than a formal certainty-of-evidence grading system. Management recommendations were evaluated separately according to whether they were label-directed, guideline/consensus-based, or pragmatic expert practice.

## 3. ADC Architecture and Mechanistic Basis of Organ Toxicity

An ADC is not simply an antibody carrying chemotherapy. Its clinical behavior is created by interactions among the target, antibody, linker, payload, and host. Target expression on malignant and normal tissues can create on-target/off-tumor toxicity. Premature linker cleavage or deconjugation can increase systemic payload exposure. Conversely, highly stable linkers may reduce extracellular exposure but require efficient internalization and intracellular processing. Membrane-permeable payloads can diffuse from antigen-positive cells to adjacent cells, producing a bystander effect that improves activity in heterogeneous tumors but may also broaden tissue injury [1,2,3,4,5,6].

T-DM1 contains the HER2-directed functions of trastuzumab and delivers DM1, a maytansinoid microtubule inhibitor, through a non-cleavable thioether linker. After HER2 binding and internalization, lysosomal degradation releases a charged lysine-MCC-DM1 catabolite with limited membrane permeability. This design produces little bystander killing and relatively limited systemic payload diffusion. Pulmonary toxicity is uncommon, and pooled cardiac analyses generally show low rates of clinically significant left ventricular dysfunction, although prior and concurrent cardiovascular exposures remain relevant [8,14,15,16,17].

T-DXd contains a humanized anti-HER2 antibody, an enzymatically cleavable linker, and a membrane-permeable exatecan-derived topoisomerase I inhibitor. Its high DAR, linker cleavage in the tumor microenvironment, and bystander effect contribute to activity in tumors with heterogeneous or low HER2 expression [3,4,5]. The mechanism of T-DXd-related ILD is incompletely defined. Nonclinical studies suggest that target-independent uptake by pulmonary immune cells, particularly alveolar macrophages, followed by intracellular payload release may contribute. Direct injury to alveolar epithelium, inflammatory amplification, impaired repair, and host susceptibility probably interact. The absence of a comparably large ILD signal with T-DM1 supports a role for the specific linker-payload configuration rather than the trastuzumab backbone alone [18,19,20,21].

SG is composed of a humanized anti-TROP-2 antibody linked to SN-38, the active metabolite of irinotecan, through a hydrolysable CL2A linker. The design permits both intracellular and extracellular payload release and produces a bystander effect. Its dominant toxicities—neutropenia, diarrhea, nausea, fatigue, alopecia, and anemia—are consistent with systemic and tissue exposure to SN-38 [9,10,11,12,22,23,24]. A direct biological explanation for a clinically meaningful cardiomyopathy signal has not been established. Rare pneumonitis may represent true drug-induced lung injury in selected cases, but hypersensitivity, infection, tumor progression, prior thoracic radiotherapy, pulmonary embolism, and toxicity from combination partners must be carefully excluded.

**Figure 1:**
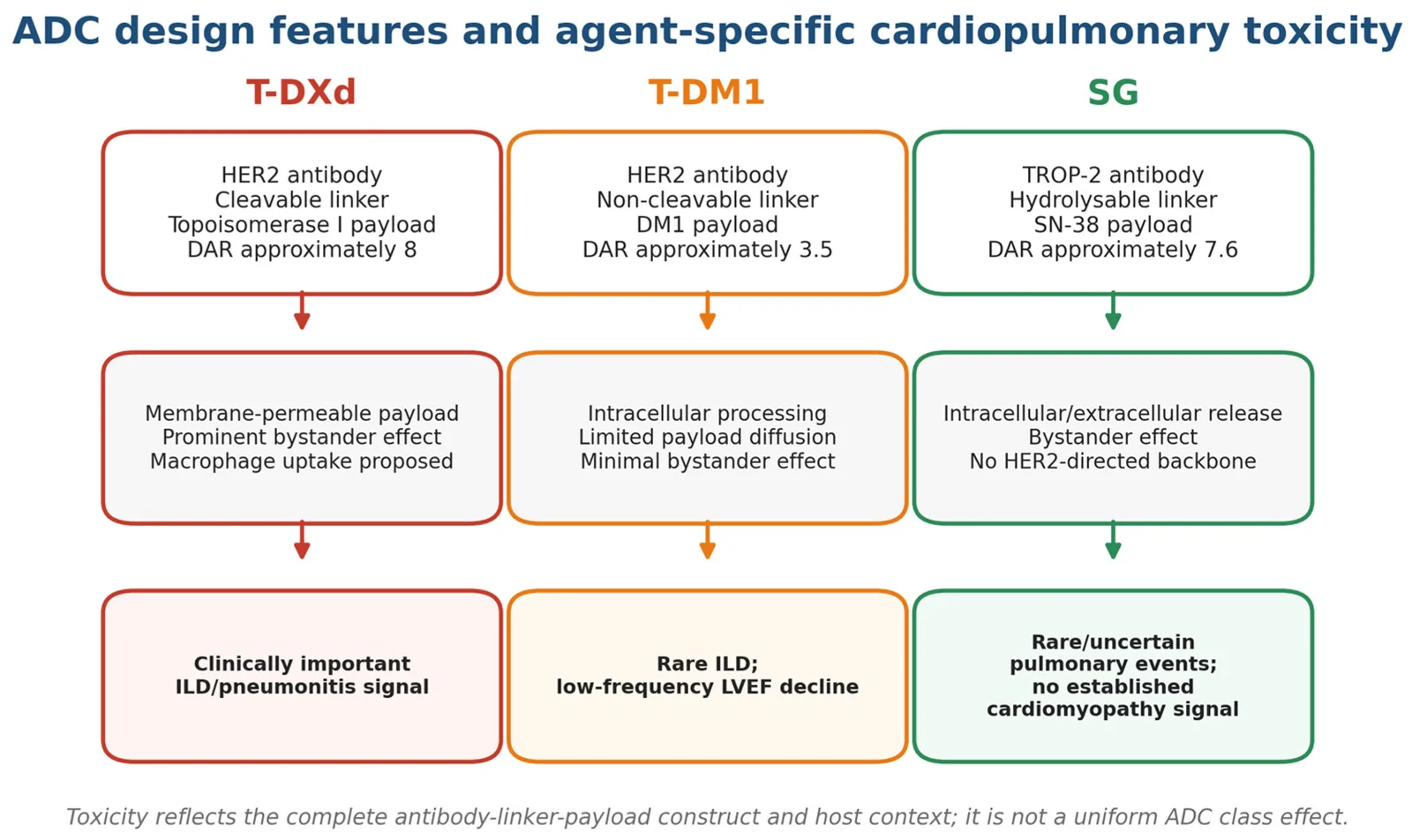
ADC design features and the mechanistic rationale for agent-specific cardiopulmonary toxicity. The diagram summarizes differences in antibody target, linker, payload, drug-to-antibody ratio, payload permeability, and bystander effect. Proposed pulmonary mechanisms for T-DXd remain incompletely defined and should not be interpreted as proven causal pathways. Arrows indicate the progression from ADC design to mechanistic features and the corresponding clinical safety signal; red, orange, and green identify T-DXd, T-DM1, and SG, respectively. DAR, drug-to-antibody ratio; ILD, interstitial lung disease; LVEF, left ventricular ejection fraction; SG, sacituzumab govitecan; T-DM1, trastuzumab emtansine; T-DXd, trastuzumab deruxtecan.

These mechanistic distinctions support a central principle: monitoring intensity should follow the known risk of the individual ADC rather than a generic ADC label. T-DXd warrants proactive pulmonary surveillance and immediate evaluation of subtle respiratory changes. T-DM1 warrants standard clinical pulmonary vigilance and structured cardiac monitoring. SG requires careful symptom-based evaluation, but current evidence does not justify importing the complete T-DXd ILD surveillance and management pathway into routine SG monotherapy. The comparative features are summarized in Table 1 and Figure 1.

## 4. Interstitial Lung Disease and Pneumonitis

Drug-induced ILD is an umbrella term encompassing heterogeneous inflammatory and fibrotic injuries of the lung parenchyma. In oncology trials, the terms ILD and pneumonitis are often grouped as an adverse event of special interest. Clinical manifestations range from asymptomatic radiographic changes to cough, dyspnea, fever, hypoxemia, acute respiratory failure, and death. CT patterns may include organizing pneumonia, hypersensitivity pneumonitis, nonspecific interstitial pneumonia, diffuse alveolar damage, and mixed or indeterminate appearances [19,25,26]. Neither symptoms nor radiology are pathognomonic; diagnosis remains one of exclusion.

### 4.1. T-DXd: Incidence, Severity, and Time to Onset

Early T-DXd development established ILD as a clinically important toxicity. In DESTINY-Breast01, which enrolled heavily pretreated patients with HER2-positive metastatic breast cancer, adjudicated drug-related ILD occurred in approximately 15% of patients, including fatal cases [27,28]. Subsequent trials used the approved breast-cancer dose of 5.4 mg/kg, strengthened education and adjudication, and introduced more explicit interruption and corticosteroid algorithms. These measures reduced the proportion of fatal events but did not eliminate ILD.

In DESTINY-Breast03, T-DXd was compared directly with T-DM1 after prior trastuzumab and taxane. ILD/pneumonitis remained more common with T-DXd, but the initial analysis did not show grade 4 or 5 adjudicated events. With longer exposure and follow-up, cumulative incidence increased, emphasizing that risk persists beyond the first months and that crude incidence is partly exposure-dependent [29,30,31]. DESTINY-Breast04 confirmed an approximately 12% incidence in HER2-low metastatic breast cancer, with a small number of fatal events [32]. The current 2026 prescribing information separately reports ILD in 12% of patients receiving T-DXd 5.4 mg/kg as monotherapy (median time to onset, 5.5 months; fatal outcomes, 0.9%), in 12% receiving T-DXd plus pertuzumab (median time to onset, 8.0 months; fatal outcomes, 0.5%). These are regimen-specific descriptive populations rather than randomized comparisons of pulmonary toxicity.

The earlier pooled analysis of nine T-DXd monotherapy studies found adjudicated drug-related ILD/pneumonitis in 15.4% of 1150 patients; most cases were grade 1 or 2, and most first events occurred within 12 months [18]. A more recent dose-stratified pooled analysis across nine DESTINY studies included 1678 patients and reported adjudicated drug-related ILD in 12.0% of the 5.4 mg/kg pool and 10.9% of the 6.4 mg/kg pool, with grade 3 or higher events in 1.8% and 1.7%, respectively [33]. These figures should not be interpreted as evidence that the higher dose is safer: the dose pools differed substantially in tumor type, demographics, renal function, prior treatment, exposure, and sample size. A breast-cancer systematic review and single-arm meta-analysis reported an overall ILD incidence of 11.7% [34]. Indication, dose, regimen, ethnicity, prior treatment, duration of therapy, CT frequency, event definitions, central adjudication, and follow-up all influence observed rates.

The clinically relevant message is not that ILD always occurs early, but that the first year is the highest-yield period for vigilance while later events remain possible. Grade 1 disease may be discovered incidentally on restaging CT. Grade 2 disease is symptomatic but not necessarily hypoxemic. Severe cases may evolve quickly, particularly when recognition, interruption, and corticosteroid treatment are delayed.

### 4.2. T-DXd: Risk Factors and Patient Selection

No single validated model reliably predicts T-DXd-associated ILD. Signals reported across pooled and real-world analyses include Japanese enrollment or Asian ancestry, dose above 5.4 mg/kg, baseline oxygen saturation below 95%, moderate or severe renal impairment, pre-existing lung disease, smoking history, longer time from initial cancer diagnosis, and pulmonary comorbidity [18,19,20,21,35,36,37]. Prior thoracic radiotherapy, lung metastases, lymphangitic disease, prior pneumonitis, and previous immune checkpoint inhibitor exposure are clinically plausible modifiers, although associations are inconsistent and confounding is substantial.

Risk factors should therefore guide discussion and surveillance rather than be used as automatic exclusion criteria. A patient with stable chronic lung disease may still derive major benefit from T-DXd, whereas active or unresolved ILD/pneumonitis generally warrants avoidance. Baseline respiratory symptoms and CT abnormalities should be characterized before treatment so that subsequent changes are interpretable. Pulmonology review is reasonable when baseline findings are unexplained, extensive, symptomatic, or likely to complicate recognition of treatment-related injury.

Renal dysfunction deserves particular attention because exposure to the payload or its metabolites may be altered and because pooled analyses identified moderate/severe renal impairment as a potential risk factor. The label recommends closer monitoring in this population [7]. However, the available evidence does not support routine prophylactic corticosteroids or exclusion based solely on age, controlled smoking history, or stable prior radiotherapy changes.

### 4.3. T-DXd: Clinical and Radiologic Presentation

Patients and caregivers should be instructed to report new or worsening cough, dyspnea, fever, chest tightness, reduced exercise tolerance, unexplained fatigue, or oxygen desaturation immediately rather than waiting for the next infusion. At each visit, a brief directed respiratory assessment is more useful than relying on spontaneous reporting. Resting oxygen saturation provides an accessible baseline and follow-up measure but cannot exclude early ILD.

High-resolution chest CT is the key imaging test when ILD is suspected. The most frequently described pattern is organizing pneumonia, often with bilateral peripheral or peribronchovascular ground-glass opacities and/or consolidations. Nonspecific interstitial pneumonia and hypersensitivity pneumonitis patterns also occur. Diffuse alveolar damage is less common but is associated with severe clinical presentations and poor outcomes [19,25,26]. Radiographic abnormalities can precede symptoms, supporting careful review of routine staging CT rather than treating the lung windows as incidental.

The differential diagnosis includes bacterial, viral, and opportunistic infection; pulmonary edema; pulmonary embolism; malignant progression; lymphangitic carcinomatosis; pleural disease; radiation pneumonitis; immune checkpoint inhibitor pneumonitis; aspiration; alveolar hemorrhage; and exacerbation of pre-existing ILD. The diagnostic pathway should be adapted to severity. Complete blood count, inflammatory markers, microbiologic testing, cardiac evaluation, CT pulmonary angiography, bronchoscopy, and bronchoalveolar lavage may be appropriate when they can clarify competing diagnoses without delaying treatment. Bronchoscopy is not mandatory in every low-grade case but is valuable in atypical, severe, immunocompromised, or infection-suspected presentations [19,25,26].

### 4.4. T-DXd: Management and Rechallenge

T-DXd should be interrupted immediately when drug-related ILD is suspected; diagnostic evaluation and treatment should proceed in parallel. Waiting for complete diagnostic certainty before interruption is unsafe. For grade 1 disease, the product label requires interruption until complete resolution and advises considering corticosteroids as soon as ILD/pneumonitis is suspected, but it does not mandate a specific grade 1 dose [7]. If resolution occurs within 28 days from onset, treatment may be resumed at the same dose; if resolution takes longer than 28 days, resumption at one dose level lower is recommended [7]. The commonly used prednisone-equivalent dose of at least 0.5 mg/kg/day, particularly for extensive, progressive, recurrent, or otherwise concerning radiographic disease, and a taper of at least four weeks derive primarily from multidisciplinary guidance and expert consensus rather than comparative management trials [19,20,21].

For grade 2 or higher ILD/pneumonitis, permanent discontinuation of T-DXd is label-directed [7]. The label also calls for prompt corticosteroid treatment; the detailed regimens used in practice are supported mainly by multidisciplinary guidance and expert consensus. Grade 2 disease is commonly treated with prednisone or equivalent at least 1 mg/kg/day until clinical improvement, followed by a taper of at least four weeks. Grade 3 or 4 disease requires hospitalization, urgent pulmonary and infectious-disease evaluation, oxygen or ventilatory support as needed, and high-dose intravenous corticosteroids; commonly proposed regimens include methylprednisolone 500–1000 mg/day for three days followed by at least 1 mg/kg/day of prednisone equivalent with a prolonged taper [7,19,20,21]. Empiric antimicrobials are reasonable when infection cannot be excluded. Lack of improvement within 48–72 h should trigger diagnostic reassessment and specialist consideration of additional immunosuppression.

Rechallenge is restricted to patients whose event remained grade 1 and resolved completely. The 2025 pooled retreatment analysis demonstrated that retreatment can be feasible, but ILD recurred in approximately one-third of retreated patients [38]. This finding supports individualized shared decision-making rather than routine rechallenge. The expected oncologic benefit, alternative therapies, extent and pattern of the first event, time to resolution, corticosteroid requirement, pulmonary reserve, and patient preference should all be considered. Rechallenge after grade 2 or higher disease remains contraindicated under current labeling. A grade- and agent-specific management framework is presented in Table 2.

### 4.5. T-DM1-Associated Pulmonary Toxicity

Pulmonary toxicity with T-DM1 is uncommon but recognized. In metastatic breast cancer datasets summarized in the current label, pneumonitis occurred in 0.8% of 884 treated patients, with one grade 3 case; the overall incidence in EMILIA was 1.2% [8]. In KATHERINE, label-defined pneumonitis was reported in 1.1% of T-DM1-treated patients, while the primary publication reported a broader pneumonitis category of 2.6% [8,39]. Radiation pneumonitis occurred in 1.8% of patients receiving adjuvant radiotherapy with T-DM1 in the label dataset, including grade 3 events [8]. Differences between publication and label categories illustrate why adverse-event definitions must be specified. Metastatic experience from EMILIA and TH3RESA further supports the low but recognized pulmonary signal [40,41].

Patients with dyspnea at rest due to advanced malignancy or comorbidity and those receiving concurrent pulmonary radiotherapy may be at increased risk. Case reports describe organizing pneumonia or interstitial pneumonitis improving after T-DM1 withdrawal and corticosteroids [42,43]. Because events are rare, validated risk factors and optimal steroid regimens are not defined. The product information recommends permanent discontinuation when ILD or pneumonitis is diagnosed. For radiation pneumonitis in the adjuvant setting, permanent discontinuation is recommended for grade 3 or higher disease or grade 2 disease that does not respond to standard treatment [8]. This uncommon but documented signal is also consistent with the broader literature on HER2-targeted therapy–associated pneumonitis [44].

The differential diagnosis is particularly important because many T-DM1 recipients have previous taxane, trastuzumab, pertuzumab, anthracycline, and radiotherapy exposure. Pulmonary embolism, infection, cardiac dysfunction, malignant progression, and radiation injury may be more likely than direct T-DM1 toxicity. Nevertheless, low incidence should not become diagnostic dismissal.

### 4.6. SG-Associated Pulmonary Events

The pulmonary safety profile of SG differs fundamentally from that of T-DXd. In ASCENT, one SG-treated patient (0.4%) developed grade 3 pneumonitis in the setting of lung metastases, previous radiation-related fibrosis, bronchial stenosis, and other major pulmonary confounders. The event resolved after permanent discontinuation, intravenous corticosteroids, and antibiotics [10,45]. In TROPHY-U-01, a grade 2 treatment-related serious ILD event was reported and resolved after SG discontinuation [46]. The current US prescribing information reports that ILD accounted for 1.1% of permanent discontinuations in ASCENT-03; this figure should not be misreported as a formally adjudicated overall ILD incidence [9].

Post-marketing disproportionality analyses have detected pneumonitis-related terms, but they cannot establish causality or incidence [11,12,23]. A 2026 case report described grade 3 interstitial pneumonitis considered probably related to SG after exclusion of competing causes and response to corticosteroid treatment [13]. These data justify clinical awareness while remaining insufficient to define a reproducible ILD syndrome comparable with T-DXd.

No SG-specific evidence supports scheduled high-resolution CT solely for ILD surveillance during monotherapy. Routine tumor-assessment CT should still be reviewed carefully, and new respiratory symptoms require prompt evaluation. During or shortly after infusion, pneumonitis-like respiratory findings must be distinguished from hypersensitivity or infusion-related reactions, which may include dyspnea, wheezing, hypotension, angioedema, and, rarely, severe cardiopulmonary compromise [9]. In SG combinations with immune checkpoint inhibitors, attribution becomes more complex because checkpoint inhibitor pneumonitis has an established independent incidence and different rechallenge considerations.

No product-label or prospectively validated SG-specific ILD algorithm is currently available. Therefore, withholding SG during evaluation of clinically meaningful unexplained pulmonary findings, excluding infection and other common causes, and using corticosteroids for probable, clinically significant drug-induced pneumonitis represent pragmatic expert practice rather than an SG-specific regulatory requirement. Permanent discontinuation is reasonable after severe or convincingly SG-related ILD, but the decision should be individualized according to causality, severity, recovery, alternative therapies, and any combination partner. This evidentiary distinction should be explicit in clinical protocols. Selected pulmonary safety evidence is summarized in Table 3.

## 5. Cardiotoxicity

Cardiotoxicity in ADC trials is reported heterogeneously. Outcomes may include asymptomatic LVEF decline, left ventricular systolic dysfunction, symptomatic heart failure, arrhythmia, QT prolongation, ischemic events, myocarditis, hypertension, or composite cardiovascular adverse events. These outcomes should not be combined indiscriminately: a protocol-defined LVEF decline detected during scheduled echocardiography is clinically different from symptomatic heart failure, and QT prolongation is not equivalent to cardiomyopathy. For consistency with the available evidence and product-label management thresholds, the principal cardiac focus of this review is cancer therapy-related cardiac dysfunction, particularly LVEF decline and heart failure. Other cardiovascular phenotypes are discussed only when an agent-specific signal or a relevant diagnostic implication is supported.

The 2022 European Society of Cardiology (ESC) cardio-oncology guideline defines cancer therapy-related cardiac dysfunction using symptoms, LVEF, global longitudinal strain, and biomarkers, and recommends baseline cardiovascular risk assessment before potentially cardiotoxic therapy [47]. Risk is shaped by pre-existing cardiovascular disease, hypertension, diabetes, dyslipidemia, obesity, smoking, age, previous anthracycline exposure, prior HER2-directed therapy, thoracic radiotherapy, and baseline ventricular function. These host and treatment factors often contribute more to absolute risk than the ADC alone. Current cardio-oncology risk assessment and management should also integrate established ESMO and HFA–ICOS recommendations [48,49]. Clinical severity and treatment-emergent adverse events remain graded using CTCAE version 5.0 [50].

The unequal length of the pulmonary and cardiac sections reflects the evidence base and clinical phenotype rather than an assumption that cardiac safety is unimportant. T-DXd-associated ILD has a drug-specific, potentially fatal syndrome with dedicated adjudication and grade-based management, whereas ADC cardiac datasets are dominated by surveillance-detected LVEF changes, heterogeneous composite terms, and few symptomatic events. The cardiac analysis therefore emphasizes phenotype, absolute event frequency, ascertainment, reversibility, competing cardiovascular causes, and regimen context rather than reproducing the pulmonary section’s structure or volume.

Across the available trial, pooled, and regulatory datasets, arrhythmias, QT prolongation, ischemic events, myocarditis, and hypertension have not formed a reproducible, dominant syndrome specific to T-DM1, T-DXd, or SG [7,8,9,16,17,34,47,51]. Such events remain clinically important and should be evaluated according to phenotype and baseline cardiovascular risk, but isolated reports or broad treatment-emergent categories should not be interpreted as proof of direct ADC causality. ECG monitoring, ischemic evaluation, or rhythm surveillance should therefore be symptom- and risk-directed unless required by a combination partner or local protocol.

A 2026 JACC: CardioOncology primer similarly emphasized that cardiovascular reporting across ADC trials remains inconsistent and that absolute clinically meaningful events should be separated from protocol-detected LVEF changes [51]. Across the reviewed evidence, symptomatic heart failure and grade 3 or higher cardiac dysfunction remain substantially less frequent than asymptomatic LVEF decline. This distinction is maintained in the agent-specific discussion and summarized quantitatively in the cardiac comparison presented below.

### 5.1. Cardiac Effects of T-DM1

T-DM1 retains the trastuzumab antibody and can therefore interfere with HER2-dependent cardiomyocyte survival signaling. However, clinical trials and pooled analyses consistently show a low incidence of important cardiac dysfunction. A pooled analysis of 1961 patients across T-DM1 trials reported a total cardiac event rate of 3.37%, driven largely by asymptomatic LVEF decline; grade 3–4 congestive heart failure or LVEF reduction occurred in 0.71%, and grade 1–2 LVEF decline in 2.04% [14]. Older age and lower baseline LVEF were associated with greater risk.

EMILIA reported LVEF reduction in a small minority of T-DM1-treated patients, and clinically significant heart failure was rare [40]. In KATHERINE, cardiac adverse events were also uncommon, although trial eligibility selected patients with preserved baseline LVEF after neoadjuvant therapy [39]. A real-world series of 41 patients identified one major LVEF decline, ultimately attributed to coronary artery disease with recovery after revascularization rather than direct T-DM1 cardiotoxicity [15]. This example illustrates the importance of investigating alternative cardiovascular causes rather than automatically assigning causality to the ADC.

A 2025 systematic review and single-group meta-analysis estimated the pooled incidence of LVEF decrease with T-DM1 at 1.09%, falling to 0.94% after adjustment for potential publication bias [17]. These data support a favorable cardiac profile but should not be interpreted as absence of risk. Pivotal trials generally excluded patients with significant cardiac disease or reduced baseline LVEF, and real-world populations may have greater cumulative anthracycline exposure, obesity, hypertension, coronary disease, or previous trastuzumab-related dysfunction.

### 5.2. Cardiac Effects of T-DXd

T-DXd also contains a trastuzumab-derived antibody and carries regulatory warnings for left ventricular dysfunction. Across breast-cancer trials, most reported cardiac events have been asymptomatic LVEF reductions; symptomatic heart failure is uncommon [7,16,17,29,30,31,32,34]. The current product information reports left ventricular dysfunction in 4.6% of the pooled 5.4 mg/kg monotherapy population, including grade 3 or 4 events in 0.6% [7]. The 2023 breast-cancer meta-analysis estimated decreased LVEF at 1.95% and reported QT prolongation separately, although definitions and surveillance varied [34]. A 2025 comparative meta-analysis estimated LVEF decrease at 4.20% with T-DXd and 0.94% with T-DM1 after adjustment [17].

The apparent difference between T-DXd and T-DM1 should be interpreted cautiously. It is an indirect cross-study comparison affected by exposure duration, line of therapy, prior treatments, baseline risk, imaging schedule, and event definition. T-DXd recipients may remain on therapy longer because of high efficacy, increasing cumulative opportunity to detect asymptomatic decline. Conversely, trial populations exclude many patients with clinically significant cardiac disease and baseline LVEF below 50%, limiting generalizability.

Contemporary T-DXd combination strategies require separate interpretation. In DESTINY-Breast09, first-line T-DXd plus pertuzumab improved progression-free survival compared with taxane-trastuzumab-pertuzumab [52]. In the corresponding regulatory safety population (*n* = 431), LVEF decrease was reported in 11% of patients, including grade 3 or 4 events in 2.1%, whereas the pooled T-DXd 5.4 mg/kg monotherapy population was reported using a different dataset and event term [7]. This difference cannot be attributed to T-DXd alone or used as an indirect estimate of excess risk: the populations, exposure, surveillance, treatment setting, and event definitions differed, and pertuzumab itself is a HER2-directed agent with recognized left ventricular effects. Cardiac event frequencies from T-DXd plus pertuzumab, T-DXd followed by taxane-trastuzumab-pertuzumab, or investigational combinations should therefore not be extrapolated automatically to T-DXd monotherapy. Baseline assessment and surveillance must follow the complete regimen and the locally applicable product information.

Potential mechanisms include HER2 blockade in cardiomyocytes, prior anthracycline-induced vulnerability, and possibly payload-related effects. A direct contribution from the deruxtecan payload has not been conclusively established. The practical implication is continued surveillance rather than a claim that T-DXd produces frequent clinical heart failure. Cardiac symptoms—dyspnea, edema, orthopnea, chest pain, palpitations, syncope, and unexplained fatigue—must also be distinguished from ILD, anemia, pulmonary embolism, infection, and cancer progression. The broader T-DXd safety database also includes gastric cancer and HER2-mutant non-small-cell lung cancer, reinforcing indication- and dose-specific interpretation [53,54].

### 5.3. Cardiac Events with SG

SG is not HER2-directed, and pivotal trials have not demonstrated a consistent syndrome of left ventricular dysfunction or heart failure. No grade 3 or higher cardiovascular toxicity was reported as a characteristic safety signal in the ASCENT safety analyses [10,45]. The dominant risks remain neutropenia, diarrhea, infection, anemia, and infusion-related reactions [9,10,22,23,24,45]. These toxicities can indirectly stress the cardiovascular system through sepsis, hypovolemia, electrolyte abnormalities, tachycardia, hypotension, and demand ischemia. Across ASCENT, TROPiCS-02, TROPHY-U-01, and earlier refractory mTNBC experience, no reproducible cardiomyopathy syndrome has emerged [45,46,55,56].

FAERS analyses identified a disproportionality signal for increased heart rate, but not an established cardiomyopathy syndrome [11]. Such signals are hypothesis-generating: they do not prove causality and may reflect anemia, dehydration, infection, pain, anxiety, prior anthracycline exposure, or advanced disease. Severe hypersensitivity may include hypotension, bronchospasm, and cardiac arrest, but this represents an acute infusion-related mechanism rather than chronic myocardial dysfunction [9].

Routine serial LVEF monitoring solely because a patient receives SG monotherapy is not mandated by current product information. Cardiac testing should instead be driven by baseline cardiovascular disease, prior cardiotoxic exposure, symptoms, clinical findings, and concomitant therapies. In practice, many patients with metastatic breast cancer will already have an indication for baseline cardiovascular assessment because of their treatment history, even when SG itself is not the principal concern.

## 6. Baseline Assessment and Surveillance

Before T-DXd or T-DM1, baseline assessment should include cardiovascular history, blood pressure, examination for congestion, review of prior anthracycline and HER2-directed exposure, thoracic radiotherapy, and measurement of LVEF by echocardiography or multigated acquisition scan. Echocardiography is generally preferred because it provides valve, chamber, and hemodynamic information and can incorporate global longitudinal strain. The ESC recommends baseline echocardiography for patients receiving HER2-targeted therapy and risk-adapted surveillance during treatment [47]. Product labels require LVEF assessment before treatment and at regular intervals [7,8].

For pulmonary risk, the baseline staging CT should be deliberately reviewed for interstitial abnormalities, radiation changes, infection, pleural disease, lymphangitic spread, and tumor burden. Respiratory history should document smoking, chronic obstructive pulmonary disease, asthma, previous ILD/pneumonitis, prior checkpoint inhibitor toxicity, thoracic radiotherapy, oxygen requirement, and current cough or dyspnea. Baseline pulse oximetry is useful; pulmonary function tests are not mandatory for every patient but can assist when pre-existing lung disease or unexplained symptoms are present.

During T-DXd treatment, respiratory symptoms should be assessed at every visit. Routine cancer restaging CT provides repeated opportunities for early detection, but the optimal CT interval for ILD surveillance has not been established and should remain consistent with disease setting and clinical risk. Any new pulmonary opacity should be actively reconciled rather than automatically attributed to infection or progression. Patients need clear instructions to contact the oncology team between cycles.

During T-DM1, symptom-directed pulmonary monitoring is usually sufficient, with particular attention during concurrent or recent radiotherapy. Cardiac monitoring should follow the product label and risk-adapted cardio-oncology guidance. During SG monotherapy, neither scheduled ILD-specific CT nor serial LVEF testing is routinely required solely because of the drug; surveillance should be individualized according to symptoms, comorbidity, previous treatment, and combination partners.

Cardiac biomarkers such as troponin and natriuretic peptides may help in high-risk patients, in those previously exposed to anthracyclines, or when symptoms or imaging changes arise. They should not be interpreted in isolation because renal dysfunction, sepsis, pulmonary embolism, tachyarrhythmia, and advanced cancer can elevate them. A consistent imaging modality and laboratory method improves longitudinal interpretation. The corresponding baseline and surveillance approach is summarized in Table 4, which distinguishes label-directed requirements from guideline/consensus-based and pragmatic recommendations. In particular, serial LVEF assessment for T-DXd and T-DM1 is label-directed, whereas the use of GLS or biomarkers, the intensity of CT review, and surveillance for SG are risk-adapted recommendations supported by guidance or pragmatic practice rather than uniform regulatory mandates. Table 5 subsequently summarizes the cardiac outcomes and surveillance definitions reported in representative major datasets.

## 7. Practical Management of Cardiac Dysfunction

For T-DXd, the label permits continuation when LVEF is above 45% with an absolute 10–20 percentage-point decline from baseline, while recommending interruption and repeat assessment within three weeks for more substantial reductions. Confirmed LVEF below 40% or an absolute decline greater than 20 percentage points requires permanent discontinuation, as does symptomatic congestive heart failure [7]. Exact thresholds should be checked against the locally applicable product information because regulatory wording may differ by jurisdiction and indication.

T-DM1 has indication-specific LVEF modification rules. In metastatic disease, treatment is withheld for LVEF below 40%, or for LVEF 40–45% with a decrease of at least 10 percentage points from baseline, with repeat assessment and discontinuation if recovery criteria are not met. Adjuvant thresholds are more conservative [8]. These rules should be incorporated into the institutional protocol rather than reconstructed from memory at the point of care.

When asymptomatic dysfunction is detected, repeat echocardiography should confirm the finding and evaluate measurement variability. Global longitudinal strain, troponin, natriuretic peptides, electrocardiography, and ischemic evaluation may clarify the phenotype. Cardio-oncology consultation is particularly valuable for symptomatic patients, LVEF below 50%, progressive decline, major biomarker elevation, previous cardiotoxicity, or limited oncologic alternatives. Angiotensin-converting enzyme inhibitors or angiotensin receptor blockers, beta-blockers, mineralocorticoid receptor antagonists, and sodium-glucose cotransporter 2 inhibitors should be used according to the clinical heart-failure phenotype and contemporary cardiology guidance.

Reliance on LVEF alone may miss earlier, subclinical treatment-related changes. In a prospective study of 35 women receiving anthracycline-containing chemotherapy for breast cancer, Özbay et al. reported early changes in echocardiographic strain and electrocardiographic repolarization parameters, and the accompanying editorial emphasized multimodal assessment beyond conventional imaging [57,58]. These publications concern anthracycline-associated cardiotoxicity rather than ADCs and therefore do not establish ADC-specific surveillance thresholds. Nevertheless, they support a phenotype-based approach that integrates symptoms, ECG, global longitudinal strain, biomarkers, LVEF, prior anthracycline exposure, and the complete anticancer regimen when early cardiac injury is suspected.

The decision to interrupt a highly effective anticancer therapy should balance cardiovascular severity, reversibility, cancer status, and alternatives. For mild asymptomatic cancer therapy-related cardiac dysfunction, contemporary cardio-oncology practice increasingly supports treatment continuation in selected patients under close specialist monitoring. However, product-specific mandatory discontinuation thresholds remain controlling. Multidisciplinary documentation should state whether the decision follows the label, a guideline-supported individualized approach, or a justified exception.

## 8. Comparative Clinical Interpretation

Applying the interpretive framework defined in Section 2, the strength of evidence is asymmetric across agents and outcomes. T-DXd-associated ILD/pneumonitis is well established by regulatory warnings, prospective trials, pooled adjudicated analyses, and real-world cohorts, although its precise incidence varies with dose, indication, exposure, surveillance, and adjudication [7,18,27,28,29,30,31,32,38]. T-DM1-associated ILD is also regulatorily recognized but uncommon, so estimates are less precise and management evidence is largely label- and consensus-based [8,41]. For SG, a reproducible drug-specific ILD syndrome remains unconfirmed; isolated trial events, case reports, and pharmacovigilance findings are best regarded as hypothesis-generating after competing causes are excluded [9,10,11,12,13,45].

The cardiac evidence requires an equally graded interpretation. Protocol-detected LVEF decline with T-DM1 and T-DXd is an established, generally low-frequency treatment-associated outcome that justifies label-directed surveillance, but clinically overt heart failure is much rarer and incompletely reported as a separate endpoint [7,8,14,17,51]. The apparently higher LVEF-event frequency in some T-DXd datasets, particularly combination regimens, is probable evidence of regimen- and ascertainment-dependent risk rather than proof of a uniformly greater intrinsic cardiomyopathy liability than T-DM1 [7,17,51,52]. For SG, absence of a reproducible cardiomyopathy pattern across pivotal trials argues against an established signal, whereas spontaneous reports of tachycardia or increased heart rate remain hypothesis-generating and vulnerable to confounding by anemia, infection, dehydration, pain, and prior therapy [9,10,11,45,46,51,55,56].

The three ADCs should not be ranked using a single composite “cardiopulmonary toxicity” measure. T-DXd has a clear, exposure-relevant ILD risk and a lower but meaningful LVEF-decline risk. T-DM1 has uncommon ILD and generally low cardiotoxicity but retains formal pulmonary and cardiac warnings. SG has rare pulmonary events and no established cardiomyopathy syndrome; most cardiopulmonary presentations during treatment are more likely to arise from indirect effects or competing diagnoses.

Cross-trial percentages can mislead for four reasons. First, exposure duration differs: an effective drug given longer accumulates more opportunities for toxicity. Second, adjudication differs: independent ILD committees identify events that investigators may miss or classify differently. Third, eligibility criteria select against baseline cardiopulmonary disease. Fourth, competing mortality and treatment discontinuation alter cumulative incidence. Real-world studies increase generalizability but introduce missing data, nonstandard surveillance, and confounding.

**Figure 2:**
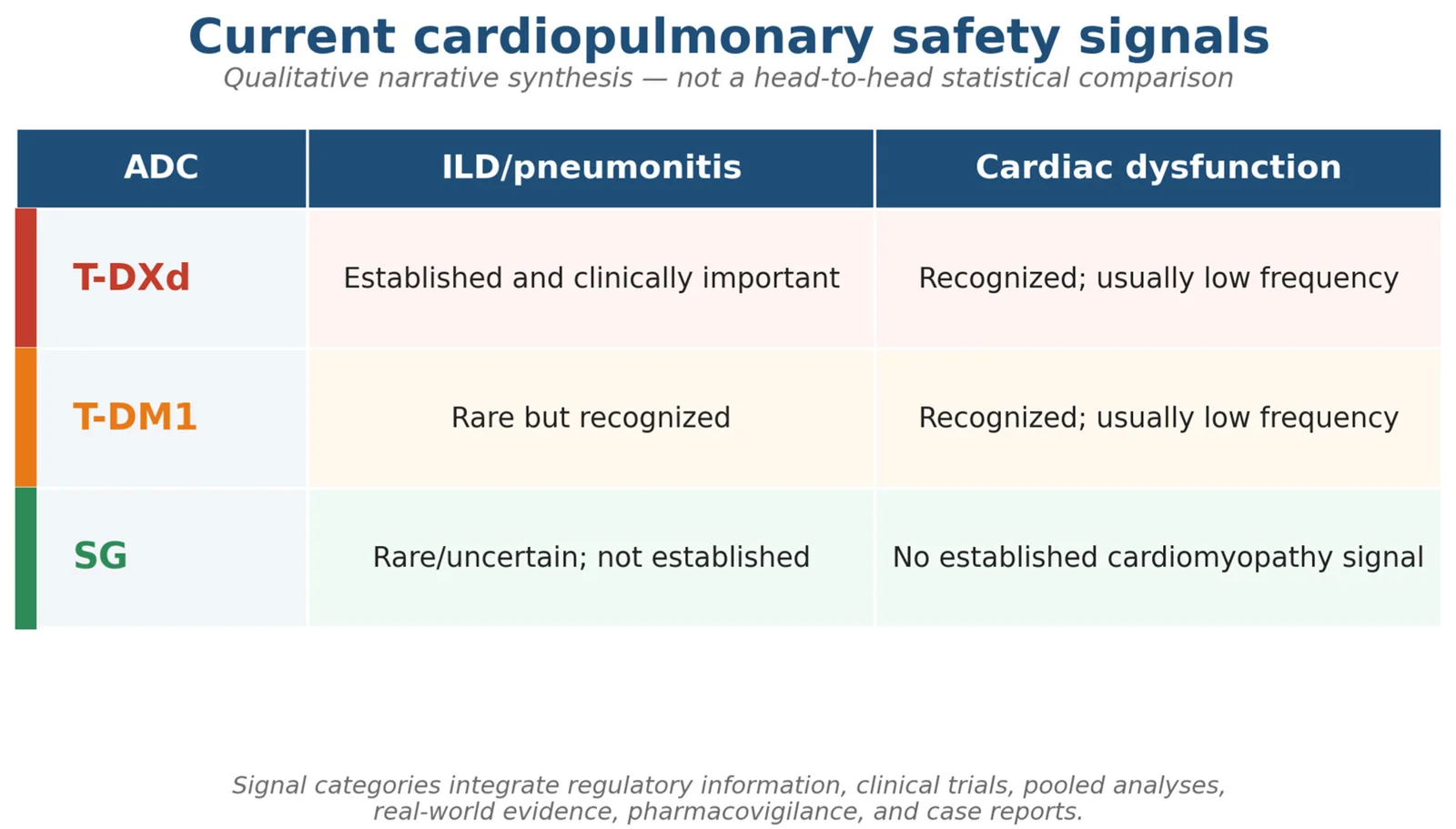
Qualitative comparison of current pulmonary and cardiac safety signals. The categories integrate regulatory information, trials, pooled analyses, real-world evidence, pharmacovigilance, and case reports reviewed in this article. Cell color and wording denote the strength and clinical prominence of the signal. The figure is a deliberately simplified clinical summary and does not represent pooled incidence estimates, duplicate the mechanistic content of Table 1, or provide a head-to-head statistical comparison.

The safest clinical approach is therefore asymmetric. T-DXd requires proactive screening, patient education, deliberate CT review, immediate interruption for suspicion, and a low threshold for corticosteroids. T-DM1 requires awareness of rare ILD, special attention around radiotherapy, and structured LVEF monitoring. SG requires standard clinical vigilance and prompt evaluation of symptoms, but not a T-DXd-equivalent surveillance program in otherwise low-risk patients. Figure 2 is deliberately retained as a simplified, decision-facing synthesis of the relative clinical prominence and evidentiary strength of the two safety signals; unlike Table 1, it does not repeat pharmacologic design features or provide quantitative incidence estimates.

Accordingly, the greater space devoted to pulmonary toxicity is clinically and evidentially justified by the mature, potentially fatal T-DXd ILD signal. Cardiac safety nevertheless requires a distinct analytical approach centered on absolute symptomatic events, standardized cancer therapy-related cardiac dysfunction phenotypes, surveillance definitions, combination partners, and alternative causes. This asymmetry should not be interpreted as lower clinical importance, but as a consequence of different event frequencies, evidence maturity, and management frameworks.

## 9. Multidisciplinary Workflow

An effective institutional pathway begins before the first dose. The oncologist documents baseline pulmonary and cardiovascular risk; the radiologist identifies pre-existing interstitial and radiation changes; cardiology or cardio-oncology evaluates high-risk cardiac disease; pulmonology evaluates unresolved respiratory abnormalities; and the patient receives written symptom instructions. Responsibility for reviewing CT lung windows and responding to between-cycle symptoms should be explicit.

When ILD is suspected, the same-day priorities are treatment interruption, severity assessment, oxygen saturation, chest CT, and exclusion of urgent alternatives. Radiology should compare the distribution and morphology of new opacities with previous studies and radiation fields. Infection and pulmonary embolism should be investigated according to probability. Grade and suspected causality should be documented, but corticosteroid treatment should not be delayed in a clinically convincing case while every alternative is exhaustively excluded.

**Figure 3:**
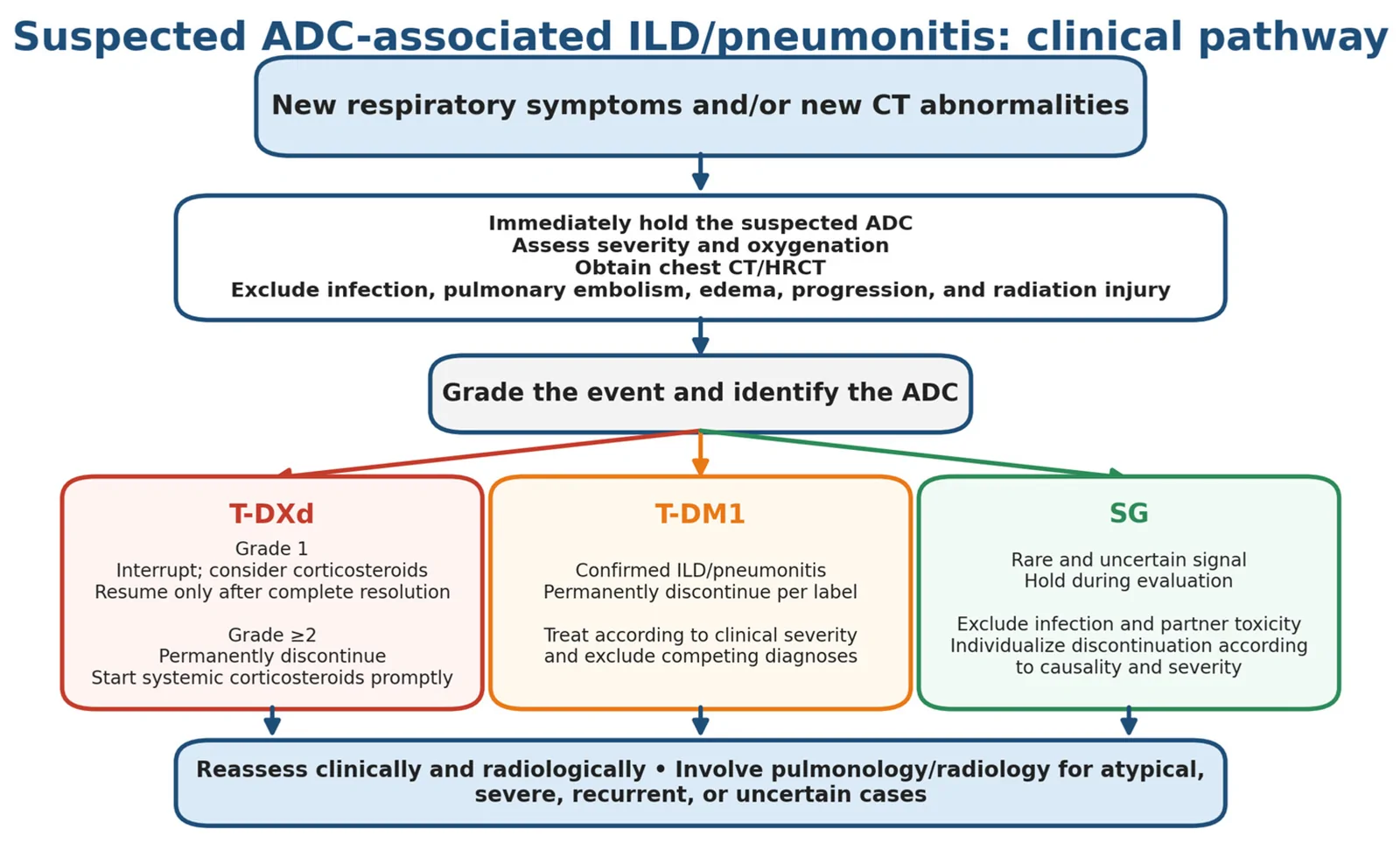
Practical pathway for suspected ADC-associated ILD/pneumonitis. The initial response is immediate treatment interruption and prompt evaluation; subsequent management must reflect the individual ADC, toxicity grade, clinical severity, competing diagnoses, and current product information. Blue arrows indicate the clinical workflow, whereas red, orange, and green identify the T-DXd, T-DM1, and SG branches, respectively. CT, computed tomography; HRCT, high-resolution computed tomography; ILD, interstitial lung disease; SG, sacituzumab govitecan; T-DM1, trastuzumab emtansine; T-DXd, trastuzumab deruxtecan.

When cardiac dysfunction is suspected, symptoms, volume status, electrocardiography, biomarkers, and echocardiography should be integrated. Dyspnea in a patient receiving T-DXd is a diagnostic fork rather than a diagnosis: ILD, heart failure, pulmonary embolism, infection, anemia, and progression may coexist. A multidisciplinary pathway reduces both dangerous under-treatment of ILD and unnecessary permanent discontinuation caused by misclassification. The practical multidisciplinary pathway is summarized in Figure 3.

## 10. Review Limitations, Evidence Gaps, and Future Directions

This review has several limitations. Its targeted narrative methodology may not have identified every relevant source, and study selection was not performed through duplicate systematic screening or a prespecified adjudication algorithm. Although overlapping trial reports were consolidated at the study-family level and final inclusion was agreed by author consensus, the process remains susceptible to subjective selection and interpretation. No formal risk-of-bias assessment or certainty-of-evidence grading was undertaken. Direct comparison among agents and regimens is constrained by heterogeneous toxicity definitions, CT and echocardiographic surveillance schedules, exposure duration, adjudication methods, follow-up, and treatment discontinuation. Breast-cancer evidence predominates, limiting generalizability to other tumor types, doses, and combinations. Pharmacovigilance databases lack validated denominators and are vulnerable to reporting bias and confounding, whereas case reports are subject to publication and attribution bias. Finally, several management recommendations rely on regulatory product information and multidisciplinary consensus rather than comparative intervention trials.

Prospective studies should harmonize ILD definitions, CT acquisition, radiologic pattern classification, adjudication, and time-to-event reporting. Crude all-grade incidence should be complemented by cumulative incidence accounting for exposure and competing events. Baseline CT abnormalities, pulmonary function, oxygen saturation, renal function, ethnicity, smoking, radiotherapy, checkpoint inhibitor exposure, and corticosteroid use should be captured consistently.

Validated predictive biomarkers are lacking. Candidate approaches include quantitative CT, radiomics, circulating inflammatory markers, pharmacogenomics, and exposure-response modeling. Artificial-intelligence-assisted CT review may improve sensitivity for subtle changes, but clinical specificity, external validation, and integration into workflow remain unresolved. A detection tool that increases false-positive interruptions could reduce therapeutic benefit.

Cardiac studies should report standardized ESC-defined phenotypes rather than isolated LVEF percentages. Global longitudinal strain, biomarkers, symptoms, heart-failure therapy, recovery, rechallenge, and cancer outcomes should be recorded. Monotherapy, sequential regimens, and combinations should be reported separately, with explicit attribution rules for each partner. The lower event rate with T-DM1 and the moderate LVEF-decline signal with T-DXd require large prospective cohorts enriched for patients commonly excluded from trials.

SG requires longer follow-up and carefully phenotyped real-world cohorts to determine whether pneumonitis and tachycardia signals represent causality, indirect effects, or reporting artifacts. Combination regimens with immune checkpoint inhibitors are particularly important because pneumonitis attribution affects discontinuation and rechallenge decisions.

Finally, ADC sequencing may modify toxicity. Prior T-DXd-related ILD could influence later treatment selection, while previous anthracycline and trastuzumab exposure modifies cardiac risk before T-DM1 or T-DXd. Registries should therefore capture the complete treatment sequence rather than analyze each ADC in isolation.

## 11. Conclusions

Pulmonary and cardiac toxicity associated with ADCs is agent-specific rather than uniform across the class. T-DXd carries a clinically important ILD/pneumonitis risk that requires proactive education, surveillance, immediate interruption for suspected disease, rapid diagnostic evaluation, and grade-directed corticosteroid treatment. Rechallenge is limited to completely resolved grade 1 events and carries a meaningful recurrence risk. T-DM1 has a substantially smaller but established pulmonary signal and generally favorable cardiac safety, while still requiring label-directed LVEF monitoring and discontinuation for confirmed ILD/pneumonitis. SG is dominated by hematologic and gastrointestinal toxicity; pulmonary events are rare and a direct cardiomyopathy signal has not been established.

The practical lesson is to avoid both complacency and overgeneralization. Rare toxicity should not be ignored, but the intensive T-DXd pathway should not automatically be applied to every ADC. Monitoring should reflect the individual drug, regimen, indication, dose, exposure, patient comorbidity, previous treatment, radiotherapy, and combination partner. Close collaboration among oncology, radiology, pulmonology, and cardio-oncology remains the most reliable strategy for preserving both treatment efficacy and patient safety.

## Figures and Tables

**Table 1:** Comparative pharmacologic features and cardiopulmonary safety profiles of T-DXd, T-DM1, and SG.

Feature	T-DXd	T-DM1	SG
Target/payload	HER2/topoisomerase I inhibitor	HER2/DM1 microtubule inhibitor	TROP-2/SN-38
Linker/approximate DAR	Cleavable tetrapeptide/~8	Non-cleavable thioether/~3.5	Hydrolysable CL2A/~7.6
Bystander effect	Prominent	Minimal	Present
Dominant established toxicities	ILD/pneumonitis, nausea, cytopenias	Thrombocytopenia, hepatotoxicity, neuropathy	Neutropenia, diarrhea, nausea
Pulmonary signal	Established; clinically important; occasionally fatal	Uncommon but label-recognized	Rare and incompletely defined
Cardiac signal	Protocol-defined LVEF decline occurs; symptomatic HF uncommon	Low incidence; mostly asymptomatic LVEF decline	No established cardiomyopathy syndrome

**Table 2:** Practical management framework for suspected or confirmed ADC-associated ILD/pneumonitis. The recommendation basis is identified as label-directed, consensus/guidance-based, or pragmatic expert practice.

Grade/Scenario	Immediate Action	Corticosteroid Approach	ADC Disposition
Suspected T-DXd ILD, grade not yet established *Basis: Label-directed interruption; consensus-supported diagnostic and steroid approach*	Interrupt T-DXd; urgent CT and exclusion of infection, PE, edema, progression	Start early when clinical/radiologic suspicion is meaningful; do not wait for exhaustive certainty	No dose until diagnosis and grade clarified
T-DXd grade 1 *Basis: Label-directed interruption/dose disposition; steroid dose from consensus guidance*	Interrupt until complete radiologic resolution; close reassessment	Consider prednisone-equivalent ≥ 0.5 mg/kg/day, especially if extensive/progressive; taper ≥ 4 weeks	Resume same dose if resolved ≤28 days; reduce one level if >28 days
T-DXd grade 2 *Basis: Label-directed permanent discontinuation; steroid dose from consensus guidance*	Permanently discontinue; pulmonology/infectious-disease input	Prednisone-equivalent ≥ 1 mg/kg/day, then slow taper ≥ 4 weeks	Permanent discontinuation
T-DXd grade 3–4 *Basis: Label-directed permanent discontinuation; intensive regimen from consensus guidance*	Hospitalize; oxygen/ventilatory support; broad diagnostic work-up	IV methylprednisolone 500–1000 mg/day for 3 days, then ≥1 mg/kg/day with prolonged taper	Permanent discontinuation
Confirmed T-DM1 ILD/pneumonitis *Basis: Label-directed discontinuation; severity-based steroid treatment*	Stop and investigate competing causes	No validated agent-specific regimen; treat according to severity	Permanent discontinuation per label; radiation-pneumonitis exceptions are indication-specific
Suspected SG pneumonitis *Basis: Pragmatic expert practice; no SG-specific regulatory ILD algorithm*	Hold during evaluation; exclude infection, infusion reaction and combination-partner toxicity	Treat clinically significant probable drug-induced pneumonitis according to severity	Individualized; severe or convincing SG-related ILD generally favors discontinuation

**Table 3:** Selected evidence describing pulmonary toxicity with the three ADCs.

Evidence Source	Population/Exposure	Pulmonary Finding	Interpretive Point
T-DXd pooled monotherapy analysis [18]	1150 patients; multiple solid tumors and doses	Adjudicated drug-related ILD/pneumonitis 15.4%; mainly grade 1–2	Incidence varies by dose, population, exposure, and adjudication
DESTINY-Breast01 [27,28]	184 heavily pretreated HER2-positive mBC patients; 5.4 mg/kg	Approximately 15% ILD; fatal events reported	Early experience before mature risk-mitigation pathways
DESTINY-Breast04 [32]	HER2-low mBC; 5.4 mg/kg	Approximately 12% ILD; small number of fatal events	Confirmed risk outside HER2-positive disease
T-DM1 regulatory datasets [8]	MBC and residual early HER2-positive breast cancer	Pneumonitis generally ~1%; radiation pneumonitis also reported	Rare, but confirmed diagnosis generally requires discontinuation
ASCENT [10,45]	Pretreated mTNBC; SG vs. physician’s-choice chemotherapy	One grade 3 pneumonitis event in SG arm with major confounders	No T-DXd-like trial signal
SG pharmacovigilance/cases [11,12,13]	Spontaneous reports and isolated cases	Pneumonitis-related signals and rare probable cases	Hypothesis-generating; cannot determine incidence or causality

**Table 4:** Agent-specific baseline assessment and surveillance. Each recommendation is identified as label-directed, guideline/consensus-based, or pragmatic expert practice; mixed categories indicate that different components of the same recommendation have different evidentiary sources.

Domain	T-DXd	T-DM1	SG
**Baseline pulmonary assessment**	Respiratory history, review baseline CT, pulse oximetry; pulmonology if abnormalities are unresolved *Basis: Consensus/guidance-based; baseline CT review is not a label-mandated test*	Clinical history and CT review; attention to radiotherapy *Basis: Pragmatic risk assessment; product information does not mandate baseline chest CT*	Clinical history; investigate baseline abnormalities according to context *Basis: Pragmatic risk assessment; no SG-specific baseline pulmonary testing requirement*
**Pulmonary surveillance**	Directed symptom screen every visit; deliberate review of each staging CT *Basis: Label-directed symptom monitoring plus consensus/guidance-based CT review*	Symptom-directed; additional vigilance with radiotherapy *Basis: Label-aware, symptom-directed pragmatic surveillance*	Symptom-directed; no routine T-DXd-equivalent ILD program *Basis: Pragmatic expert practice; no SG-specific ILD surveillance algorithm*
**Baseline cardiac assessment**	LVEF before therapy; cardiovascular risk assessment *Basis: Label-directed LVEF assessment plus guideline-based cardiovascular risk assessment*	LVEF before therapy; cardiovascular risk assessment *Basis: Label-directed LVEF assessment plus guideline-based cardiovascular risk assessment*	Risk-based; not mandated solely by SG monotherapy *Basis: Pragmatic, risk-based assessment; not required solely by the SG label*
**Cardiac surveillance**	Serial LVEF at regular intervals; risk-adapted biomarkers/GLS *Basis: Label-directed serial LVEF; GLS/biomarkers are guideline- and risk-based*	Serial LVEF at regular intervals *Basis: Label-directed serial LVEF; additional testing is guideline- and risk-based*	Driven by prior therapy, comorbidity, symptoms and combination partners *Basis: Pragmatic, risk- and symptom-based surveillance*
**Key diagnostic trap**	Dyspnea may be ILD, HF, PE, infection, anemia or progression *Basis: Pragmatic differential-diagnosis framework*	Dyspnea may reflect HF, PE, infection, radiation injury or rare ILD *Basis: Pragmatic differential-diagnosis framework*	Pulmonary symptoms may reflect infection, infusion reaction, disease or checkpoint-inhibitor pneumonitis *Basis: Pragmatic differential-diagnosis framework*

**Table 5:** Cardiac outcomes and surveillance definitions in representative major datasets. Event terminology and ascertainment differed across studies; NR indicates that symptomatic heart failure or grade-specific outcomes were not reported separately from a composite or broader cardiac category.

ADC/Study	Population/Regimen	All-grade LVEF Outcome	Symptomatic HF/Grade ≥ 3 Events	Surveillance/Event Definition
T-DM1 pooled analysis [14]	Seven trials; *n* = 1961 (T-DM1, *n* = 1544; T-DM1 + pertuzumab, *n* = 417)	Grade 1–2 LVEF decline: 2.04%	Symptomatic HF: not separable from the composite; grade 3–4 CHF/LVEF decline: 0.71%	Pooled protocol-defined cardiac events: CHF/grade 3–4 LVEF decline, ischemia, arrhythmia, or grade 1–2 LVEF decline
T-DM1: EMILIA and KATHERINE [8,39,40,51]	EMILIA, *n* = 991 overall; KATHERINE, *n* = 1486 overall	Left ventricular dysfunction: 1.8% in EMILIA; 0.4% in KATHERINE	Symptomatic HF: rare and not consistently separated; EMILIA: one grade 3 LVEF event; KATHERINE: 0.1% adjudicated cardiac events (not a grade-specific LVEF endpoint)	Protocol-scheduled LVEF; trial/label-defined left ventricular dysfunction and adjudicated cardiac events
T-DXd: DESTINY-Breast03 [29,30,31,51]	*n* = 524 overall; T-DXd versus T-DM1	LVEF decrease: 2.3% with T-DXd; 0.4% with T-DM1	Symptomatic HF: NR separately; grade ≥ 3: NR separately	Protocol-scheduled LVEF; trial-defined LVEF decrease
T-DXd: DESTINY-Breast04 [32,51]	*n* = 557 overall; T-DXd versus physician’s choice	Grade ≥ 2 LVEF decrease: 13.4% (grade 2, 11.9%; grade 3, 1.5%)	Symptomatic HF: NR separately; grade 3 LVEF decrease: 1.5%	Protocol-scheduled LVEF; CTCAE-graded LVEF decrease
T-DXd monotherapy [7]	Current pooled regulatory safety population; 5.4 mg/kg	Left ventricular dysfunction: 4.6%	Symptomatic HF: NR separately; grade 3–4: 0.6%	Regulatory composite term; baseline and serial LVEF assessment
T-DXd + pertuzumab: DESTINY-Breast09 [7,51,52]	Regulatory safety population; *n* = 431	LVEF decrease: 11%	Symptomatic HF: NR separately; grade 3–4: 2.1%	Regulatory term ‘LVEF decrease’; results cannot be extrapolated to monotherapy
SG: ASCENT and TROPiCS-02 [10,45,51,55]	ASCENT, *n* = 529; TROPiCS-02, *n* = 543	No cardiac events reported as a reproducible trial signal	Symptomatic HF or grade ≥ 3 dysfunction: none reported as a characteristic signal	No SG-specific serial LVEF surveillance paradigm; symptom- and risk-directed assessment

## Data Availability

No new data were generated or analyzed in this study. All information is derived from previously published studies, which are cited within the manuscript.

## Abbreviations

The following abbreviations are used in this manuscript:

ADC	Antibody–drug conjugate
CT	Computed tomography
CTCAE	Common Terminology Criteria for Adverse Events
DAR	Drug-to-antibody ratio
ESC	European Society of Cardiology
FAERS	FDA Adverse Event Reporting System
HF	Heart failure
HER2	Human epidermal growth factor receptor 2
ILD	Interstitial lung disease
LVEF	Left ventricular ejection fraction
mBC	Metastatic breast cancer
mTNBC	Metastatic triple-negative breast cancer
PE	Pulmonary embolism
SG	Sacituzumab govitecan
T-DM1	Trastuzumab emtansine
T-DXd	Trastuzumab deruxtecan
TROP-2	Trophoblast cell-surface antigen 2
